# Case Report: Bilateral Biportal Endoscopic Open-Door Laminoplasty With the Use of Suture Anchors: A Technical Report and Literature Review

**DOI:** 10.3389/fsurg.2022.913456

**Published:** 2022-06-07

**Authors:** Chengyue Zhu, Jing Wang, Wei Cheng, Dong Wang, Hao Pan, Wei Zhang

**Affiliations:** ^1^ Department of Orthopaedics, Hangzhou Traditional Chinese Medicine Hospital Affiliated to Zhejiang Chinese Medical University, Hangzhou, China; ^2^ Department of Orthopaedics, Hangzhou Ding Qiao Hospital, Hangzhou, China

**Keywords:** bilateral biportal endoscopy, cervical laminoplasty, suture anchor, endoscopic spine surgery, cervical stenosis

## Abstract

**Background:**

Unilateral biportal endoscopy (UBE) is a newly developed technique for spine surgery. Owing to the convenience of nerve decompression and compatibility with open surgical instruments under endoscopic guidance, this technique has seen widespread global use. In this study, we first used modified UBE with suture anchor fixation for cervical laminoplasty in a 65-year-old female patient with good clinical outcomes.

**Methods:**

We used bilateral biportal endoscopy (BBE) for cervical laminoplasty with suture anchor fixation in a patient with cervical stenosis. Under endoscopic guidance, a bilateral approach was used to make the gutter and lift the lamina door. After the lamina doors were opened, sutures were tied tightly using facia cannula and knot pusher. After confirming the solidarity of the open-door status, the drainage tube was inserted and the incisions were closed. The patient’s pre- and postoperative radiological and clinical results were evaluated.

**Results:**

Postoperative Japanese Orthopaedic Association (JOA) and Neck Disability Index (NDI) scores were improved clinically, and cervical canal was decompressed radiologically.

**Conclusions:**

BBE laminoplasty combined with suture anchor fixation showed a favorable clinical and radiological result and appears to be a safe and effective technique for cervical stenosis.

## Introduction

With the advent of an aging society, an increasing number of patients with cervical stenosis require decompression surgery (1). Traditional open surgery has many disadvantages (2) hence, minimally invasive approaches to surgery have emerged, which lead to less injury, faster recovery, and fewer complications (3). Unilateral laminectomy with bilateral decompression in a narrow cervical canal with percutaneous endoscopy and microscopy is risky and has a steep learning curve (4–6). Unilateral biportal endoscopy (UBE) is a minimally invasive spinal surgery developed in recent years, which has been widely used for degenerative diseases of the lumbar spine with remarkable efficacy (7–11). UBE for cervical decompression has also been reported, but there have been only a few published studies (12, 13). In a technical report, we applied a modified UBE technique for cervical laminoplasty with the aid of suture anchor fixation, and obtained satisfactory clinical results.

## Materials and Methods

### Ethics Statement and Case Presentation

**Figure 1:**
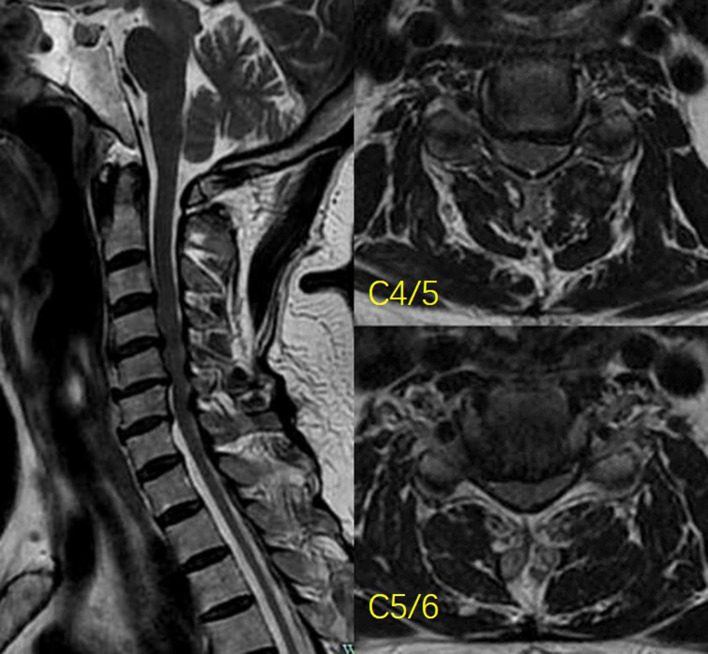
Preoperative MR images show central canal stenosis at the C4-C6. The spinal cord was compressed by herniated discs and a hypertrophied ligamentum flavum.

The study approval was obtained from our institutional review board (NO. 2019KY006). Informed consent was obtained from the patient for the publication of the report. A 65-year-old woman was suffering from gait disfunction and numbness in both the upper extremities for 5 years, and her condition deteriorated within 1 month. A physical examination revealed tendon hyperreflexia and the presence of a Babinski sign in the lower extremities with positive Hoffman sign and hypoesthesia in the upper extremities (more severe on the right side). The patient’s history included controlled hypertension and hyperlipidemia. Magnetic resonance imaging (MRI) revealed central cervical stenosis at the C4-C5-C6 levels, and the spinal cord was compressed due to ligamentum flavum (LF) hypertrophy and disc herniation at the C4-C5-C6 levels (Figure 1). The diagnosis of cervical myelopathy was confirmed. The patient expressed strong opposition to conventional open surgeries, such as anterior fusion and posterior open-door laminoplasty because of a history of cardiovascular diseases and agreed to biportal endoscopic cervical laminoplasty. The Japanese Orthopaedic Association (JOA) score was 9 and Neck Disability Index (NDI) score was 23 when she visited the outpatient department.

### Procedure

#### Position, Incision, and Instruments

**Figure 2:**
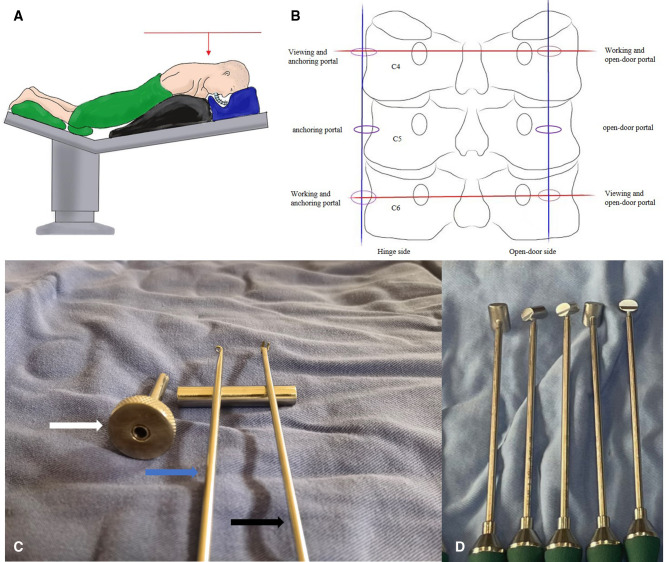
(**A**) A overall view of operating table. The table was adjusted to make sure that the intervertebral space was perpendicular to the ground. (**B**) Schematic representation of the location of the portals; (**C**) Newly designed knotting system for endoscopic laminoplasty. Facia cannula (white arrow), working length 6 cm and inner diameter 0.5 cm; endoscopic knot pusher (blue arrow) and shears (black arrow), diameter 0.3 cm; (**D**) Trials (range, 8 mm–12 mm) for the measurement of LOS.

Under general anesthesia, the patient was placed prone on a spine table with the head secured in a horseshoe headrest. The cervical spine was mildly flexed and fixed by a tape (Figure 2A).

Two horizontal lines were drawn along the C4 and C6 pedicles, a vertical line in the midline of the right lateral mass, while the other one was along the lateral margin of the left lateral mass in the anteroposterior view. The intersection points on the left-hand side served as a viewing portal, whereas the intersection points on the right-hand side served as a working portal. Two more incisions were made at the midpoints of the intersections on both sides. The three incisions on the left were used as the anchoring portal while the contralateral incisions were used as the open-door portal (Figure 2B).

We used suture anchors with a length of 10 mm and diameter of 2.8 mm. A high-speed burr (Guizhou Zirui Technology, China), specially designed arthroscopic facilities (Figure 2C), a tool-kit of radiofrequency (RF) systems (Jiangsu BONSS Medical Technology, China), and open spine surgical instruments, including pituitary forceps, curettes, and Kerrison rongeurs, were used.

#### Bilateral Biportal Approach for Laminoplasty

**Figure 3:**
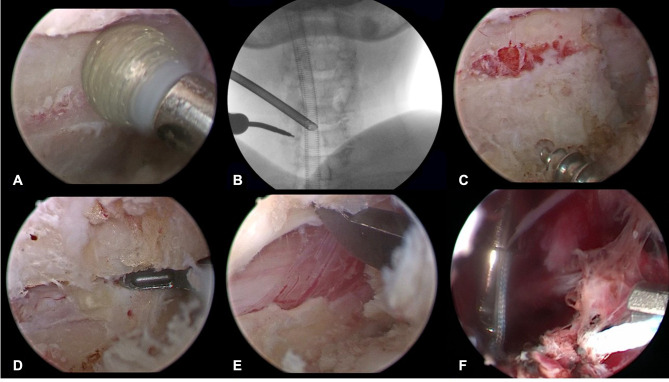
The drilled gutter was first completed using 4-mm diamond burr on the hinge side (**A**) Insertion hole for the suture anchor was also prepared using 2-mm diamond burr on the hinge side and confirmed under fluoroscopy (**B**) Suture anchor was screwed in the lateral mass (**C**) The remained ventral cortical bone on the open-door side was resected using 1-mm Kerrison rongeur (**D**) The lamina door was raised by a 2-mm Kerrison rongeur (**E**) The sutures was knotted tightly to maintain the lamina door in open position (**F**).

The first stage of the procedure was performed on the left side of the patient. A RF was used to expose the lamina, spinous process, and lateral mass of C4-C5-C6. A 4-mm diamond burr was used to remove the dorsal cortex and cancellous bone at the junction of the lamina and lateral mass of C4-C5-C6 (Figure 3A), and the bony gutter on the hinge side was completed. Subsequently, a 2-mm diamond burr was employed to make entry points on the center of the lateral mass and spinous processes of C4-C5-C6 (Figure 3B); suture anchors were inserted in succession (Figure 3C).

The endoscopy and instruments were moved to the right to begin the second stage of the procedure. After C4-C5-C6 lamina and intersections of C34 and C67 were exposed, the same longitudinal gutter was created at the margin between the lamina and the lateral mass of C4-C5-C6 using a 4-mm diamond burr. The remaining ventral cortex was removed by a 1 mm Kerrison rongeur with a thin blade (Figure 3D). The LF between C34 and C67 was cut transversely to facilitate the process of floating laminae.

**Figure 4:**
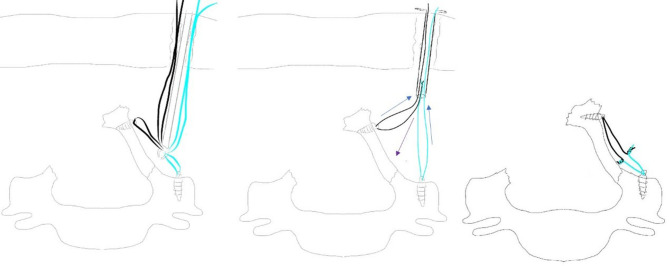
Schematic representation of knotting process.

At the beginning of the third stage, the tip of spinous process of C4-C5-C6 was carefully cut. The arthroscopy was passed over the top of the lamina via the interspinous ligament, reaching the contralateral side to observe the knotting process. A retriever was used to take the previously introduced sutures for each segment out of the same soft tissue portal; then, the facia cannula was inserted along the sutures in the anchoring portal. The assistant bent the hinge in a greenstick fashion and held the lamina door in place (Figure 3E). The sutures were tied firmly to prevent reclosure of the lifted laminae (Figures 3F, 4).

#### Drainage and Closure

A drainage tube was inserted on the open-door side, and the incisions were closed using a standard method (Supplementary Video S1). The surgery was performed without complications. The estimated blood loss during the operation was 200 mL, and the operation time was 190 min. Cefuroxime (1.5 g, twice a day, intravenous) was administered for 24 h, and the pain was managed with flurbiprofen (100 mg, twice a day, intravenous) postoperatively.

A postoperative semirigid cervical collar was prescribed for 3 months. The patient gave consent to the regular anti-osteoporosis treatment which could effectively improve bone mineral density and prevent anchors from loosening and displacement.

## Results

**Figure 5:**
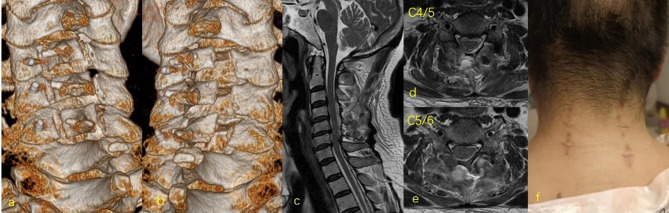
Postoperative 3D CT of C4-C6 showed widening of the spinal canal after BBE laminoplasty using suture anchor fixation (**A**,**B**) Postoperative MRI showed full decompression of spinal cord with an adequate subarachnoid space (**C–E**). Six wounds were created for the cervical laminoplasty at three levels (**F**).

Postoperative computed tomography scan revealed adequate enlargement of cervical canal of C4-C5-C6 and the cord was fully decompressed on MRI (Figure 5). The JOA and NDI scores were 11 and 20 on day 3 after surgery, and improved to 14 and 16 at the 6 months follow-up, respectively.

## Discussion

To the best of our knowledge, this is the first study to apply the UBE and suture anchor techniques for cervical laminoplasty. An endoscopic open-door laminoplasty with minimal muscle invasion was performed. The patient’s clinical symptoms improved significantly.

Cervical spondylotic myelopathy caused by posterior compression often requires posterior surgery. Open laminectomy and laminoplasty necessitate extensive paraspinal muscle release and retraction, which may result in axial pain and kyphotic deformity during follow-ups (14). In percutaneous endoscopic and microscopic surgery, contralateral decompression can be completed only when the base of spinous process is excessively removed, which leads to more intraspinal work and a higher risk. The operation and observation in a single portal manifested several difficulties (4, 6).

The UBE technique has been widely used in the treatment of lumbar degenerative diseases, with less iatrogenic injury and faster recovery (15, 16). The application of UBE in cervical spine was mainly in foraminoplasty (12, 17); There is only one report on spinal canal decompression and the technical requirements are relatively high (13). We have designed a contralateral “Zhang’s portal” to facilitate cervical canal decompression (18), therefore, we have rich experience in bilateral biportal operations. Kurokaw (19) demonstrated the validity of suture anchors in cervical laminoplasty in a cadaveric study, and we successfully performed suture anchor fixation on the contralateral side using the UBE technique. Compared with open surgery, continuous irrigation not only ensures a clear operative field, but also lowers the risk of infection (7, 20). The working portal is independent of the viewing portal, and thus the operative efficacy is dramatically improved. Moreover, under the arthroscope, the field of vision is enlarged 30 times, and the observation of dural pulsation, tiny blood vessels, and ligaments is very clear, which is conducive for hemostasis and decompression.

Before the operation, we considered placing the plate on the open-door side, however, it was very difficult to operate under the arthroscope. Without specially designed instruments, it was also difficult to find the tiny internal fixation materials once they were lost in the soft tissue. Besides, the cervical canal was opened and the spinal cord exposed, which was vulnerable to internal fixation. Suture anchor repair is technically mature under arthroscopy (21). Our team has a lot of experience in arthroscopic surgery and suture management, so we chose the suture anchor fixation on the hinge side under UBE guidance. The indications of this procedure were the same as those of traditional open cervical laminoplasty, while the contraindications included severe osteophytes around the lateral mass and abnormally distributed vessels which make safe anchor placement difficult, and prior cervical surgery with posterior approach.

There are some tips that surgeons should not overlook. The lateral mass should be fully exposed on the hinge side, but not on the open-door side. Exposure beyond the lateral margin of the lateral mass will significantly increase bleeding. Guttering should be done before anchoring; otherwise, the sutures can easily get tangled. When creating the open side, the ventral cortex of the laminae and LF were removed carefully using a 1 mm Kerrison rongeur, a nerve hook was used to separate the adhesions, and a low frequency probe was used to decrease the bleeding from epidural veins. No force was applied during the open-door procedure. The lamina open-door angle should not exceed 45° (22). After opening the lamina, trials (Figure 2D) for miniplate were used to make sure that the laminoplasty opening size (LOS) was large enough for decompression (23). Before knotting, the retriever was used to retrieve the sutures to the same soft tissue portal, and then the fascia cannula was inserted along the sutures to help avoid soft tissue incarceration. Under the arthroscopic supervision, knot in the fascia cannula should be fastened to prevent lamina reclosure.

However, the described technique has some limitations. The operation should be performed by experienced surgeons and assistants. Large sample studies in multicenters should be conducted to determine further clinical outcomes. With the development of surgical instruments and techniques, plate and screw fixation on the hinge side may be a better choice.

In conclusion, we described a bilateral biportal laminoplasty for the treatment of cervical stenosis. With the innovation of endoscopic instruments and techniques, this modified technique will play a role in cervical disorders.

## Data Availability

The original contributions presented in the study are included in the article/Supplementary Material, further inquiries can be directed to the corresponding author/s.
