# Supplementary material for: Case Report: Bilateral Biportal Endoscopic Open-Door Laminoplasty With the Use of Suture Anchors: A Technical Report and Literature Review
Source: Front Surg. 2022 Jun 7;9:913456. doi: 10.3389/fsurg.2022.913456 (PMC9209651; doi:10.3389/fsurg.2022.913456)
Supplement: Supplementary file 2 [file Table_2_v1.doc]

**Reviewer 1**

I congratulate you on this innovative and successful surgery. It is remarkable that biportal technique is used to perform cervical laminoplasty.
The authors described a challenging technique of bilateral biportal endoscopic creation of gutter and laminoplasty and using suture anchor to maintain the laminoplasty door opened.
It was mentioned the knots are passed through fascia tube, please elaborate and show how the forceps passed through the fascia tube to fetch the sutures vs sutures are retrieved by retriever like shoulder scope.

Answer: Thank you for your valuable comments. As you have suggested, we have elaborated the knotting procedure in the “Materials and Methods” section.

Line 84-88

At the beginning of the third stage, the tip of spinous process of C4-C5-C6 was carefully cut. The arthroscopy was passed over the top of the lamina via the interspinous ligament, reaching the contralateral side to observe the knotting process. A retriever was used to take the previously introduced sutures for each segment out of the same soft tissue portal; then, the facia cannula was inserted along the sutures in the anchoring portal.

Please demonstrate how the assistant lift up the lamina safely to allow the knots to be tied on the opened cervical lamina. A video showing how the surgery is performed would be helpful as a supplementary file.

Answer: Thank you for this pertinent comment. A video clip was added to help the readers better understand the procedure. 

English grammar needs edition.

Answer: According to your suggestion, we made a great effort to correct the spelling and grammar errors and polish the whole manuscript.

**Reviewer 2、**

This is an interesting study of bilateral biportal endoscopic (BBE), which applied the unilateral biportal endoscopic (UBE) and suture anchors for laminoplasty. The combined technique showed favorable clinical and radiological results, which appears to be a safe and effective technique for cervical stenosis. But there are still some questions.
1. To facilitate the better understanding of this technology, please attach the intraoperative photos of surgical incision (anchoring portals and open-door portals) and overall view of operating table, as well as the postoperative neck view.

Answer: Thank you for this reasonable comment. Unfortunately, realistic pictures were lost, but we have provided schematics of intraoperative images of surgical incision and the overall view of the operating table in Fig 2c,2d. The postoperative neck view was added in Fig 5f.

2. On the anchoring portal, how to bent the hinge in a greenstick fashion, in case of complete lamina rupture.

Answer: Thank you for this reasonable comment. We have added more comments and references in the Discussion section with exact details about this issue.

line 143-145

No force was applied during the open-door procedure. The lamina open-door angle should not exceed 45° (22)

Line 228-230

[22] Shrestha D, Miao J, Zhang J, et al. Effect of Titanium Miniplate Fixation on Hinge Fracture and Hinge Fracture Displacement Following Cervical Open-Door Laminoplasty[J]. International Journal of Spine Surgery, 2020, 14(4):7061.

3. On the open-door portals, how to evaluate the best open-door degree, and how to prevent dural sac tears.

Answer: Thank you for this reasonable comment. Generally, LOS of 10–12 mm at C3–C7 might be optimal during open-door cervical laminoplasty. Trials (range, 8 mm~12 mm) in Fig 2d can be used for the measurement of laminoplasty opening size. We have added one more reference.

Line 231-234

[23] Gu Z, Zhang A, Shen Y, et al. Relationship between the laminoplasty opening size and the laminoplasty opening angle, increased sagittal canal diameter and the prediction of spinal canal expansion following open-door cervical laminoplasty[J]. European Spine Journal, 2015, 24(8):1613-1620.

As you pointed out, dural tear was one of most common complications in endoscopic spine surgery. We have proposed some advice to avoid this complication

Lines 141-143

When creating the open side, the ventral cortex of the laminae and LF were removed carefully using a 1 mm Kerrison rongeur, a nerve hook was used to separate the adhesions, and a low frequency probe was used to decrease the bleeding from epidural veins.

4. For the insufficient strength of suture anchors, how to hold the lamina door in right place and how to avoid the risk of shedding or fracture during postoperative follow-up.

Answer: Thank you for this valuable comment. Kurokaw (19) demonstrated the validity of suture anchors in cervical laminoplasty in a cadaveric study. We have added postoperative course to avoid the risks

[19] Kurokawa Y, Yokoyama Y, Kuroda K, et al. Biomechanical evaluation of the suture anchors used in open-door laminoplasty: a cadaveric study. Spine. 2014, 39(21), E1248–E1255. DOI:10.1097/BRS.0000000000000522

Lines 97-99
A postoperative semirigid cervical collar was prescribed for 3 months. The patient gave consent to the regular anti-osteoporosis treatment which could effectively improve bone mineral density and prevent anchors from loosening and displacement.

5. Maintaining stable water dynamics is the first step in creating a safe and complete environment for UBE, but in this multi-portal surgery, how to maintain the stable water dynamics?

Answer: Thank you for your reasonable comment. We sutured the temporarily useless incisions to maintain stable water dynamics.

**Reviewer 3、**

I want to congratulate the authors for producing an excellent manuscript introducing their surgical technique. More and more surgeons choose to offer options with the least side effect for the patient; this is an example of a new approach based on biportal endoscopy.
It should be mentioned that surgeons who wish to perform this type of procedure need to have microsurgical skills and especially know everything about cervical laminoplasties to later apply them with skills in spinal endoscopy, specifically biportal endoscopy.
Despite considering that this manuscript deserves to be published in the journal, I would like to make minimal consideration to the authors.

1.- A revision for native English.

Answer: Thanks for your comments, we made a great effort to correct grammar errors and polish the whole manuscript.
2.- Between 2.1 and 2.2, it would be proper to add a sentence with the surgical treatment proposed to the patient, in this case, "biportal endoscopic cervical laminoplasty."

Answer: Thank you for your valuable comment. The proposal has been added (Line 53-54).

3.- It would be very informative if the authors added the specific indications and contraindications criteria for this technique.

Answer: Thank you for this reasonable comment. We have added the criteria for indications and contraindications.

Line 134-137

The indications of this procedure were the same as those of traditional open cervical laminoplasty, while the contraindications included severe osteophytes around the lateral mass and abnormally distributed vessels which make safe anchor placement difficult, and prior cervical surgery with posterior approach.

4.- It would be great if the authors added the postop skin incisions of the patient.

Answer: Thank you for this reasonable comment. The postoperative skin incisions are shown in Fig 5f.

5.- is the last follow-up 6-mo?

Answer: The patient was followed up by telephone over 12 months. She moved far away from our city and got better over time. Unfortunately, she refused to come back for a radiological check-up.
This could be an exciting text for experts in spine surgery and minimally invasive techniques.

**Reviewer 4、**

Authors presented a technique report on bilateral biportal endoscopic Open-Door laminoplasty using suture anchors for a multi-level cervical stenosis.
This is an ingenious idea to achieve the laminoplasty under the guidance of arthroscopy and is the first well executed case reported.
The grammar and wording were slightly awkward, but does not detract from understanding and need only light editing.
But the paper still needs minor revisions prior to consider it for publication.
1. I will be better educated on how this is accomplished if their video clips were provided.

Answer: Thank you for this reasonable comment. A video clip has been added to help the readers better understand the operation. 
2. In Fig 4, how does newly designed knotting system help knot?

Answer: We have updated Fig 4 and elaborated the knotting procedure in the “Materials and Methods.”

Line 84-88

At the beginning of the third stage, the tip of spinous process of C4-C5-C6 was carefully cut. The arthroscopy was passed over the top of the lamina via the interspinous ligament, reaching the contralateral side to observe the knotting process. A retriever was used to take the previously introduced sutures for each segment out of the same soft tissue portal; then, the facia cannula was inserted along the sutures in the anchoring portal.
