# Supplementary material for: Case Report: Bilateral Biportal Endoscopic Open-Door Laminoplasty With the Use of Suture Anchors: A Technical Report and Literature Review
Source: Front Surg. 2022 Jun 7;9:913456. doi: 10.3389/fsurg.2022.913456 (PMC9209651; doi:10.3389/fsurg.2022.913456)
Supplement: Supplementary file 1 [file Table_1_v1.doc]

May 16, 2022

Jaimo Ahn

Specialty Chief Editor

*Frontiers in Surgery: Orthopedic Surgery*

Dear Editor:

We wish to re-submit the attached manuscript as an Original Article. The manuscript ID is 913456.

The manuscript has been rechecked and appropriate changes have been made in accordance with the reviewers’ suggestions. The responses to their comments have been prepared and attached herewith.

We thank you and the reviewers for your thoughtful suggestions and insights, which have enriched the manuscript and produced a better and more balanced account of the research. We hope that the revised manuscript is now suitable for publication in your journal.

Sincerely,

Wei Zhang

Department of Orthopaedics, Hangzhou Traditional Chinese Medicine Hospital Affiliated to Zhejiang Chinese Medical University, Tiyuchang Road NO. 453, Hangzhou, 310007, China

Email: [volcano8060@163.com](mailto:volcano8060@163.com); Tel: +86-13696369519
